# Both Reaction Time and Accuracy Measures of Intraindividual Variability Predict Cognitive Performance in Alzheimer's Disease

**DOI:** 10.3389/fnhum.2018.00124

**Published:** 2018-04-09

**Authors:** Björn U. Christ, Marc I. Combrinck, Kevin G. F. Thomas

**Affiliations:** ^1^Applied Cognitive Science and Experimental Neuropsychology Team Laboratory, Department of Psychology, University of Cape Town, Cape Town, South Africa; ^2^Division of Geriatric Medicine, Groote Schuur Hospital, Department of Medicine, University of Cape Town, Cape Town, South Africa

**Keywords:** Alzheimer's disease, accuracy, cognition, episodic memory, intraindividual variability, reaction time

## Abstract

Dementia researchers around the world prioritize the urgent need for sensitive measurement tools that can detect cognitive and functional change at the earliest stages of Alzheimer's disease (AD). Sensitive indicators of underlying neural pathology assist in the early detection of cognitive change and are thus important for the evaluation of early-intervention clinical trials. One method that may be particularly well-suited to help achieve this goal involves the quantification of intraindividual variability (IIV) in cognitive performance. The current study aimed to directly compare two methods of estimating IIV (fluctuations in accuracy-based scores vs. those in latency-based scores) to predict cognitive performance in AD. Specifically, we directly compared the relative sensitivity of reaction time (RT)—and accuracy-based estimates of IIV to cognitive compromise. The novelty of the present study, however, centered on the patients we tested [a group of patients with Alzheimer's disease (AD)] and the outcome measures we used (a measure of general cognitive function and a measure of episodic memory function). Hence, we compared intraindividual standard deviations (iSDs) from two RT tasks and three accuracy-based memory tasks in patients with possible or probable Alzheimer's dementia (*n* = 23) and matched healthy controls (*n* = 25). The main analyses modeled the relative contributions of RT vs. accuracy-based measures of IIV toward the prediction of performance on measures of (a) overall cognitive functioning, and (b) episodic memory functioning. Results indicated that RT-based IIV measures are superior predictors of neurocognitive impairment (as indexed by overall cognitive and memory performance) than accuracy-based IIV measures, even after adjusting for the timescale of measurement. However, one accuracy-based IIV measure (derived from a recognition memory test) also differentiated patients with AD from controls, and significantly predicted episodic memory performance. The findings suggest that both RT- and accuracy-based IIV measures may be useful indicators of underlying neuropathology. The present study therefore contributes toward an understanding of the relative utility of RT- and accuracy-based IIV measures in detecting neurocognitive impairment in older adults, and also advances the empirical evaluation of sensitive markers of cognitive change in patients with AD.

## Introduction

Twenty-three percent of the worldwide burden of disease occurs in individuals age 60 years and older, and up to 63% of individuals with age-related diseases such as dementia currently reside in low- and middle-income countries (LAMICs; Prince et al., 2015; World Health Organisation, 2015). In one such country, South Africa, the most recent census statistics indicate that 8% of the population (~4.1 million individuals) are aged 60 years or older, and that that number will increase by as much as 40% over the next two decades (Statistics South Africa, 2014). Furthermore, community-based estimates suggest there is a higher prevalence of dementia in South Africa, and in LAMICs generally, compared to global estimates (Prince et al., 2013; de Jager et al., 2015). These epidemiological data underscore the urgency of conducting LAMIC-based dementia research.

Recently, the Alzheimer's Association's Research Roundtable (AARR), an interdisciplinary group of leading dementia researchers, prioritized the urgent need for sensitive measurement tools that can detect cognitive and functional change at the earliest (even prodromal) stages of Alzheimer's disease (AD; Snyder et al., 2014). Sensitive indicators of underlying neural pathology are important for the evaluation of early-intervention clinical trials, and may play a central role in alleviating the burden of age-related disease (Food and Drug Administration, 2013). One method that may be particularly well-suited to help achieve this goal involves the quantification of *intraindividual variability* (IIV; also known as *inconsistency)* in cognitive performance. Whereas conventional indicators of cognitive performance are based on measures of central tendency and involve assessment of an individual on a single measure administered on a single occasion, IIV indicators are based on measures of variability and involve assessing fluctuations in performance of an individual on a single measure administered on multiple occasions (Li et al., 2001; Hultsch et al., 2002; MacDonald et al., 2006).

Contemporary IIV research focuses primarily on inconsistency in performance on reaction time (RT) measures (see, e.g., Bielak et al., 2010; Saville et al., 2011; Bunce et al., 2013; Yao et al., 2016). Such latency-based measures are particularly well-suited to IIV research because they have larger ranges than traditional cognitive test scores, thus making them more sensitive than traditional cognitive tests to individual performance differences. RT tasks also (a) typically involve multiple trials, which allows for many samples of performance, and (b) are less sensitive to re-test effects (Allaire and Marsiske, 2005; Salthouse, 2012). Over the past two decades, a sizeable literature has established IIV in RT as an effective marker of general cognitive function in older adults: High levels predict impending cognitive decline, and are associated with a range of age-related neurological disturbances, with neurodegenerative disease (e.g., AD), and with mortality risk (Collins and Long, 1996; Hultsch et al., 2002; MacDonald et al., 2003; Burton et al., 2006; Shipley et al., 2006; Duchek et al., 2009; Bielak et al., 2010).

An alternative method for capturing IIV involves using accuracy-based measures. These measures are derived from tasks featuring stimuli to which the test taker makes either a correct or an incorrect response (e.g., Murphy et al., 2007; Tractenberg and Pietrzak, 2011). Because such tasks are used frequently in clinical practice, deriving an IIV score from them, and showing the predictive value of that score, is a useful undertaking. Although some studies report that accuracy-based IIV measures can, for instance, differentiate between patients with AD, those with Parkinson's disease, and healthy controls, and can aid in detecting prodromal AD (Darby et al., 2002; Burton et al., 2006; Murphy et al., 2007; Tractenberg and Pietrzak, 2011; Kälin et al., 2014), many researchers prefer latency-based measures. One reason for this preference is that statistically significant positive associations between accuracy-based IIV and age do not survive after controlling for mean performance. Associations of outcomes (e.g., age, clinical group status) with RT-based IIV measures are not affected by controlling for mean performance, and hence those measures are perceived to be superior in detecting underlying pathology (see, e.g., Li et al., 2001; Salthouse et al., 2006).

However, only one previous study in the aging literature provides a direct comparison of the relative sensitivity of RT- and accuracy-based IIV measures to cognitive compromise. Hultsch et al. (2000) measured trial-to-trial and session-to-session IIV in RT- and accuracy-based measures in three groups: healthy older adults, patients with arthritis, and patients with dementia (either mild AD or mild vascular dementia). They reported that, whereas there were no significant between-group differences in terms of accuracy-based IIV, RT-based measures differentiated the groups successfully, independent of mean-level predictors.

The current study seeks to systematically replicate and extend the findings of Hultsch et al. (2000). Specifically, we also compare directly the relative sensitivity of RT- and accuracy-based estimates of IIV to cognitive status. The novelty of the present study, however, centers on the patients we tested and the measures we used. Where Hultsch et al. (2000) used a mixed-dementia group, we use a group of patients with AD. Inclusion of this more homogenous clinical group allows for improved sensitivity of accuracy-based tasks, which are typically designed to target specific domains of cognitive function (e.g., episodic memory). Furthermore, where Hultsch and colleagues' analyses were targeted toward categorical prediction of group membership (i.e., they asked whether RT- and accuracy-based IIV measures could distinguish healthy older adults from patients with arthritis and from patients with dementia), our analyses use more variable outcome measures (i.e., we ask not only whether RT- and accuracy-based IIV are significantly different in healthy older adults compared to patients with AD, but also whether those IIV measures are predictive of performance on a measure of general cognitive function and on a measure of performance in a cognitive domain that, typically, is sensitive to AD dysfunction). In summary, the specific aims of our analyses were to (a) use RT- and accuracy-based measures of IIV to differentiate between a clinical group of AD patients and a control group of demographically matched healthy individuals, (b) determine the relative contribution of RT- and accuracy-based measures of IIV to the prediction of overall cognitive functioning and episodic memory functioning, and (c) evaluate the effect of the timescale of measurement on that relative contribution of RT- and accuracy-based measures of IIV to the prediction of overall cognitive functioning and episodic memory functioning.

Hence, the present study contributes toward an understanding of the relative utility of RT- and accuracy-based IIV measures in detecting neurocognitive impairment in older adults, and also responds to the AARR call for empirical evaluation of sensitive markers of cognitive change in patients with AD.

## Materials and methods

### Design and setting

The current study is the first report of data collected within an ongoing longitudinal investigation of AD progression taking place in Cape Town, South Africa. The parent study utilizes a measurement burst design (Nesselroade, 1991), in which each participant experiences three intervals of serial testing (or bursts; T1, T2, and T3) over the course of 12 months. Within each interval, each participant is tested three times (e.g., T1.1, T1.2, T1.3) over a 2-week period. The data we report here are from the first test interval (i.e., T1).

### Participants

All participants (*N* = 48; 34 women) were over the age of 55 years (*M* = 71.25, *SD* = 7.12). The sample consisted of a cognitively healthy control group (*n* = 25; 19 women) and a mild-to-moderate stage possible or probable AD clinical group (*n* = 23; 15 women), with diagnosis following NINCDS-ADRDA criteria (McKhann et al., 1984).

Clinical participants were recruited from a state hospital's Memory Clinic. Recruitment was monitored by health professionals, including a neurologist (MIC) and a neuropsychologist (KGFT), who provide clinical service delivery at the Clinic. Control participants were community-dwelling volunteers from the greater Cape Town area. They received notice of the study via word-of-mouth or flyers distributed to seniors' clubs, old age homes, and retirement villages.

Inclusion criteria were (a) availability of medical health history; (b) age 55 years or above; (c) English literacy (i.e., basic ability to speak, read, and write in that language); and (d) availability of a close relative or similar who could provide information about recent changes in cognitive function. Exclusion criteria included (a) a diagnosis of HIV/AIDS, uncontrolled hypertension, uncontrolled diabetes mellitus, or any other medical condition that, in the opinion of the research team, might have a long-lasting effect on cognitive function; (b) current or present psychiatric illness; (c) a Geriatric Depression Scale (GDS; Yesavage et al., 1982) score > 9/30; (d) the presence of any major neurological disorder (e.g., Parkinson's disease, Huntington's disease) or past stroke; (e) any history of alcohol or drug abuse, or heavy smoking (> 20 cigarettes per day); and (f) Mini-Mental State Examination (MMSE; Folstein et al., 1975) score < 12.

The Research Ethics Committees of the University of Cape Town's Department of Psychology and Faculty of Health Sciences approved all study procedures. These procedures adhered to the guidelines published in the Declaration of Helsinki (World Medical Association, 2013).

### Measures and procedures

The data we report on here were gathered across four sessions (one screening and three test sessions). All study participants signed consent forms before screening. Moreover, all the clinical participants were informed about the study and consent was signed in the presence of a guardian/caregiver/relative (who also signed the consent form). The screening session occurred no more than 30 days before the first test session (in most cases, there was a week or less of separation). For all participants, the three test sessions took place over a 2-week period.

Sessions were held in a private research room at Groote Schuur Hospital or at the participant's home, depending on his/her preference and travel capabilities. All tests were administered by BUC, or by a graduate student trained and supervised by him.

#### Screening session

This session included administration of (a) a detailed clinical interview that gathered information about biographical, medical, and psychiatric history, (b) the GDS, (c) the MMSE, and (d) the Cambridge Examination for Mental Disorders of the Elderly-Revised edition (CAMCOG-R; Huppert et al., 1995). The latter was developed as a cognitive screening measure for the early diagnosis of dementia in the elderly (Leeds et al., 2001). It consists of 67 items and measures cognitive performance within eight domains (orientation, language, memory, attention, praxis, calculation, abstract thinking, and perception). We used a version adapted for use with South African samples (James et al., 2014).

At the conclusion of the session, participants were invited back for repeated administration of a 10-test cognitive battery (see Supplemental Material for the full list of tests). Below, we describe the five tests for which data are reported.

#### Test sessions

The order of test administration was varied for each session to prevent order effects (see Supplemental Material for the different test orders). All three test sessions were otherwise identical to one another. Each lasted ~2 h.

##### Reaction time tasks

These tasks are part of the Cambridge Neuropsychological Test Automated Battery (CANTAB; Fray et al., 1996). All CANTAB tests are administered on a touch-screen computer. On the simple reaction time (SRT) task, a yellow dot appears inside a circle placed at the center of the computer screen. Participants are required to release a press pad and touch the dot as quickly as possible after its onset. On the choice reaction time (CRT) task, the yellow dot appears inside one of five circles located on the screen. Both tests include a 10-trial practice phase that precedes the test phase. Participants are required to obtain 90% accuracy on the practice trials before proceeding to the test phase. Those who fail to achieve this criterion are presented with a second practice phase. Thereafter, they proceed to the test phase regardless. The test phase for both the SRT and the CRT tasks consists of 30 trials. A single block of trials (e.g., 30 SRT trials) takes ~5 min to complete.

We administered two SRT blocks and two CRT blocks in each session. Hence, after three test sessions and six blocks of administration we had collected data from 180 trials of SRT performance and 180 trials of CRT performance for each participant.

##### Accuracy-based tasks

We used two subtests from the Repeatable Battery for the Assessment of Neuropsychological Status (RBANS), a short screening battery for identifying and characterizing dementia in the elderly (Randolph et al., 1998). These subtests measure immediate and delayed episodic memory, a prominent domain of dysfunction in the cognitive profile of AD (Traykov et al., 2007). There are four parallel forms for each of the RBANS memory subtests, making them appropriate for repeat assessments and allowing for the tracking of cognitive decline within neurodegenerative processes (Randolph et al., 1998).

On the RBANS *List Learning* subtest, the participant is read a list of 10 words, and is instructed immediately thereafter to recall as many as possible. This process is repeated four times. After a 25–35 min delay, the participant is asked to recall the list, and immediately thereafter is administered a recognition task (i.e., to identify which words from a group of 20 (10 targets and 10 foils) were present on the original list). On the RBANS *Story Memory* subtest, the participant is read a brief story, and is instructed immediately thereafter to recall as many elements of the story as possible. This process is repeated twice. After a 25–35 min delay, the participant is asked to recall the story.

Although we administered a different form of the List Learning and Story Memory subtests at each test session, the order of administration was the same for each participant (i.e., all participants received List A and Story A at the first test session, List B and Story B at the second session, and so on).

### Statistical analyses

#### Data preparation

##### RT tasks: filtering data

We examined the RT data for outliers because unusually fast or slow responses may reflect spurious performance (e.g., temporary distraction, interruption, or fast guesses). Following convention (see, e.g., Hultsch et al., 2000; Bielak et al., 2010; Garrett et al., 2012), we removed scores that were either (a) below a lower limit for authentic responses at 150 ms, or (b) above an upper limit of 3 *SD* above the group RT mean for each block of testing. Missing data were then imputed for the outlier trials using a regression-based multiple imputations method (Lachaud and Renaud, 2011). This method of filtering the data is thought to offer conservative estimates of performance variability (e.g., Hultsch et al., 2002).

##### RBANS and CAMCOG-R tasks: deriving variables

We derived three scores from the RBANS subtests. The *List Learning* score is the sum of the number of words recalled correctly across the four learning trials (range 0–40). The *List Recognition* score is the total number of correctly identified words on the recognition trial (range 0–20). The *Story Memory* score is the sum of the number of items recalled correctly across the two learning trials (range 0–24).

We derived two scores derived from the CAMCOG-R: Total Score (assessing general cognitive function; range = 0–105), and the recent memory and learning subscale composite score (assessing episodic memory function; range = 0–21). We chose to use the latter because (a) episodic memory dysfunction is a key feature of AD (Peña-Casanova et al., 2012), and (b) the composite score is relatively resistant to the influence of education (James et al., 2014). This is an important consideration given that almost half of the variance in CAMCOG-R scores is accounted for by the effects of age and education (Pereiro et al., 2015).

##### Extracting intraindividual variability

Computing IIV scores requires an initial purification of systematic effects in the data that are explained by mean performance scores. Specifically, although one might calculate the intraindividual standard deviation (*iSD*), calculating raw *SD*s may introduce systematic effects associated with mean RT because slower mean RTs are strongly associated with higher *SD*s, and vice-versa (Hale et al., 1988; Hultsch et al., 2008). Therefore, before computing *iSD*s it is important to partial out any factors (e.g., group and time-on-task effects such as practice and fatigue) that may influence mean RT performance.

To determine which factors significantly influenced the means of RT- and accuracy-based variables, and to thus extract *iSD*s, we ran a random intercept model on the sample data for each of the SRT, CRT, List Learning, List Recognition, and Story Memory variables, and then added two sets of main effects, the first [featuring test order, blocks, trials (or sessions for the accuracy-based tasks)] to evaluate the impact of time-on-task effects, and the second (featuring group status, sex, monthly household income, age, and level of education) to evaluate the impact of group effects.

#### Inferential statistical analyses

We conducted all inferential analyses using SPSS (version 24), with α set at 0.05.

The first part of the analysis involved analyzing between-group differences in demographic, cognitive, and affective variables. We used independent-samples *t*-tests for parametric data, chi-squared tests of contingency for categorical data, Mann-Whitney *U* tests for non-parametric data, and when the assumption of homogeneity of variance for the Mann-Whitney *U* tests was not upheld we used independent-samples *t*-tests and bootstrapped 1,000 replicates using bias corrected (BCa) confidence intervals. To estimate effect sizes, we used Cohen's *d*, phi (ϕ), and *r* for *t*-tests, chi-squared tests, and Mann-Whitney *U* tests, respectively. We interpreted these effect sizes following Cohen's (1988) guidelines: for Cohen's *d*, 0.2 = small, 0.5 = moderate, 0.8 = large; for ϕ and *r*, 0.1 = small, 0.3 = moderate, 0.5 = large.

The second part of the analysis set the stage for subsequent regression modeling by examining bivariate associations (using Pearson's *r* correlation coefficient) between each candidate predictor (i.e., the *iSD* for each of the SRT, CRT, List Learning, List Recognition, Story Memory variables, and the mean for each of those variables) and each cognitive outcome variable (i.e., CAMCOG-R Total Score and CAMCOG-R Memory Composite).

The final part of the analysis involved creation of a series of sequential multiple regression models that sought to determine the relative contribution of RT- and accuracy-based IIV measures to the prediction of (a) overall cognitive functioning, and (b) episodic memory functioning.

## Results

### Sample characteristics

The groups were well matched in terms of age, sex distribution, monthly household income, and current depressive symptomatology, but there were significant between-group differences in terms of education, with participants in the control group having completed more years of formal schooling (see Table 1). As expected, the analyses also detected significant between-group differences (associated with large effect sizes) on the two CAMCOG-R outcome measures, with the control group scoring better in each case.

**Table 1:** Descriptive statistics and between-group differences: sample demographic, affective, and cognitive characteristics (*N* = 48).

**Variable**	**GROUP**		***df***	***t*/x^2^/ *z***	***p***	**ESE**
	**Control**	**AD**				
	**(*n* = 25)**	**(*n* = 23)**				
Age (years)	69.76 (7.37)	72.87 (6.63)	46	1.53	0.132	0.44
Sex (M:F)	6:19	8:15	1	0.67	0.412	−0.12
Education (years)^a^	11.80 (2.43)	10.13 (2.97)	46	−2.14	0.038^*^	0.62
Income^b^	7959.50 (4376.595)	6477.76 (4094.00)	46	1.21	0.232	0.35
GDS	5.88 (3.24)	6.74 (2.91)	46	0.96	0.341	0.28
MMSE	28.08 (1.61)	21.48 (5.13)		−4.47	<0.001^***^	0.65
CAMCOG-R						
Total Score	91.26 (5.18)	69.00 (13.45)	27.93	−7.68	<0.001^***^	2.18
Memory Composite	17.36 (1.32)	8.26 (4.62)	25.29	−9.10	<0.001^***^	2.68

For the variables Age, Education, Income, GDS, MMSE, CAMCOG-R Total Score, and CAMCOG-R Memory Composite the second and third columns present means with standard deviations in parentheses. AD, Alzheimer's disease; ESE, effect size estimates (for t, Cohen's d, for chi square, φ, and for Mann-Whitney, Cohen's r); GDS, Geriatric Depression Scale; MMSE, Mini-Mental State Examination; CAMCOG-R, Cambridge Cognitive Examination for Mental Disorders of the Elderly-Revised.

a Highest level of education attained.

b Monthly household income, in South African Rands (ZAR). At the time of the study, the US$:ZAR exchange rate was 1:13.53.

* p < 0.05,

*** *p < 0.001. All p-values are two-tailed*.

### Primary analyses

#### Extraction of iSDs

We followed the extraction approach described by Hultsch et al. (2008). Random intercept models identified the following fixed effects that contributed significantly to mean performance on each of the candidate predictors: for SRT, blocks, group status, and sex significantly predicted trial-to-trial performance; for CRT, test order, blocks, group status, and sex significantly predicted trial-to-trial performance; for List Learning, group status significantly predicted session-to-session performance; for List Recognition, group status, and session significantly predicted session-to-session recognition performance; and for Story Memory, task session, group status, sex, and education level significantly predicted session-to-session scores. (See Supplemental Material for the full set of results.)

Next, we entered, for each candidate predictor, the significant fixed effects and all their higher-order interactions into a random coefficient model with random slopes on trials (or sessions for the accuracy-based variables) in order to partial out time-on-task and group effects. Finally, we captured the residuals, converted them to *T*-scores, and calculated the *SD* across the *T*-score values to compute the *iSD*s.

#### Between-group differences: predictor variables

On both RT-based measures, *iSD* scores for controls were, on average, significantly lower than those for patients. In contrast, the same significant between-group difference was only present for one of the accuracy-based measures (List Recognition; see Table 2).

**Table 2:** Descriptive statistics and between-group differences: predictor variables (*N* = 48).

**Variable**	**Group**		***df***	***t/z***	***p***	**ESE**
	**Control**	**AD**				
	**(*n* = 25)**	**(*n* = 23)**				
***iSD***						
SRT	7.69 (1.26)	10.27 (3.18)	28.27	3.63	0.001^**^^a^	1.07
CRT	7.57 (1.33)	10.37 (3.05)	29.55	4.07	<0.001^***^	1.19
List Learning	10.29 (5.72)	10.34 (5.65)		−0.16	0.877	0.02
List Recognition	7.28 (5.35)	12.45 (6.41)		−3.21	0.001^**^	0.46
Story Memory	9.52 (5.36)	10.15 (5.34)	46	0.41	0.685	0.12
**MEAN**						
SRT	319.20 (31.32)	349.57 (62.18)	31.88	2.11	0.043^*^	0.62
CRT	351.15 (36.10)	396.21 (70.77)	32.11	2.74	0.010^*^	0.80
List Learning	26.61 (3.85)	17.70 (4.57)	46	−7.33	<0.001^***^	2.11
List Recognition	18.97 (0.91)	14.33 (2.49)	27.39	−8.43	<0.001^***^^b^	2.48
Story Memory	15.83 (2.48)	9.36 (3.85)	46	−6.97	<0.001^***^	2.00

Data presented are means, with standard deviations in parentheses. AD, Alzheimer's disease; ESE, effect size estimates (for t, Cohen's d and for Mann-Whitney, Cohen's r), iSD, intraindividual standard deviation; SRT, simple reaction time; CRT, choice reaction time.

a BCa 95% CI [1.26 to 4.04], p = 0.002;

b BCa 95% CI [−5.73 to −3.50], p = 0.001.

* p < 0.05.

** p < 0.01.

*** *p < 0.001. All p-values are two-tailed*.

Regarding mean-level performance variables, control participants achieved significantly faster reaction times on the CANTAB tasks, and performed significantly more accurately on the RBANS subtests, than patients (see Table 2).

#### Regression modeling

##### Bivariate associations between predictor and outcome variables

Among *iSD* scores, those for SRT, CRT, and List Recognition showed significant moderate-to-large negative associations with both outcome variables. Among mean scores, each predictor variable was significantly associated, with moderate-to-large magnitude and in the expected direction, with each outcome variable (see Table 3). Based on this set of findings, we excluded List Learning and Story Memory *iSD* scores from subsequent analyses.

**Table 3:** Bivariate correlations: predictor and outcome variables (*N* = 48).

**Predictor variable**	**CAMCOG-R Outcome Variable**	
	**Total score**	**Memory composite**
***iSD***		
SRT	−0.59^**^	−0.63^**^
CRT	−0.56^**^	−0.66^**^
List Learning	−0.03	−0.05
List Recognition	−0.40^**^	−0.45^**^
Story Memory	−0.13	−0.09
**MEAN**		
SRT	−0.38^**^	−0.42^**^
CRT	−0.45^**^	−0.52^**^
List Learning	0.76^**^	0.80^**^
List Recognition	0.74^**^	0.82^**^
Story Memory	0.88^**^	0.85^**^

CAMCOG–R, Cambridge Cognitive Examination for Mental Disorders of the Elderly-Revised. iSD, intraindividual standard deviation; SRT, simple reaction time; CRT, choice reaction time.

** *p < 0.01. All p-values are one-tailed*.

##### Sequential regression models

We regressed a set of demographic variables (age, sex, education, income), after controlling for group status, on each of the two CAMCOG-R outcome variables to determine which demographic factors were significant predictors of, respectively, overall cognitive functioning and episodic memory functioning. For CAMCOG-R Total Score, significant predictors were group (β = −0.72, *t* = −8.38, *p* < 0.001), sex (β = −0.27, *t* = −3.13, *p* < 0.01), and education (β = 0.27, *t* = 2.76, *p* < 0.01). For CAMCOG-R Memory composite, significant predictors were group (β = −0.80, *t* = −9.10, *p* < 0.001) and sex (β = −0.24, *t* = −2.77, *p* < 0.01).

Then, we created a set of models that described how *trial-to-trial variability* on RT tasks, relative to session-to-session variability on accuracy-based tasks, predicted (a) CAMCOG-R Total Score, and (b) CAMCOG-R Memory Composite score. For each model, we entered the significant demographic factors identified above at the first step, *iSD* RT- and accuracy-based predictors at the second, and mean-based predictors at the third (see Table 4). The purpose of taking this third modeling step was to determine if the significant *iSD* predictors identified at Step 2 would continue to make a unique contribution toward prediction of the outcome variable after controlling for (a) means of *iSD* predictors entered at Step 2, and (b) means of the List Learning and Story Memory scores (entered because they are widely-used mean-level predictors of episodic memory performance in the clinical setting).

**Table 4:** Regression models: trial-to-trial reaction time IIV compared to accuracy-based IIV in the prediction of CAMCOG scores (*N* = 48).

**Predictor**	**CAMCOG-R OUTCOME VARIABLE**			
	**Total Score**		**Memory Composite Score**	
	**Model 1**	**Model 2**	**Model 3**	**Model 4**
	**SRT vs. List Recognition**	**CRT vs. List Recognition**	**SRT vs. List Recognition**	**CRT vs. List Recognition**
**STEP 1**				
Group	−0.72^***^	−0.72^***^	−0.84^***^	−0.84^***^
Sex	−0.26^**^	−0.26^**^	−0.26^**^	−0.26^**^
Education	0.23^*^	0.23^*^		
**STEP 2**				
Group	−0.57^***^	−0.59^***^	−0.63^***^	−0.63^***^
Sex	−0.22^**^	−0.24^**^	−0.20^**^	−0.21^**^
Education	0.22^*^	0.23^*^		
***iSD***				
List Recognition	−0.11	−0.08	−0.17^*^	−0.14
SRT/CRT	−0.23^**^	−0.17	−0.29^**^	−0.28^**^
**STEP 3**				
Group	−0.31^**^	−0.28^*^	−0.33^**^	−0.29^**^
Sex	−0.15	−0.15	−0.13	−0.12
Education	0.08	0.11		
***iSD***				
List Recognition	−0.04	−0.01	−0.08	−0.06
SRT/CRT	−0.28^*^	−0.10	−0.25^*^	−0.19^*^
**Mean**				
List Learning	−0.03	−0.01	0.01	−0.02
List Recognition	−0.01	0.09	0.15	0.23
Story Memory	0.51^**^	0.47^**^	0.34^*^	0.34^*^
SRT/CRT	0.14	0.01	0.09	−0.01

Data presented are β (standard regression coefficient) values. CAMCOG-R, Cambridge Cognitive Examination for Mental Disorders of the Elderly-Revised. SRT, simple reaction time; CRT, choice reaction time.

* p < 0.05,

** p < 0.01,

*** *p < 0.001. All p-values are one-tailed*.

The most notable results at the second step were these: After controlling for demographic variables, *iSD*s for List Recognition and SRT contributed significantly to the prediction of CAMCOG-R Total Score [Model 1: Δ*R*^2^ = 0.04, *F*_(2, 42)_ = 4.23, *p* = 0.02, Cohen's *f* = 0.20], and CAMCOG-R Memory composite score [Model 3: Δ*R*^2^ = 0.08, *F*_(2, 43)_ = 8.08, *p* < 0.01, Cohen's *f* = 0.37]. *iSD*s for List Recognition and CRT contributed significantly to the prediction of Memory composite score [Model 4: Δ*R*^2^ = 0.07, *F*_(2, 43)_ = 7.31, *p* < 0.01, Cohen's *f* = 0.34]. List Recognition *iSD* score only predicted one outcome variable (viz., Memory Composite) significantly when it was entered together with SRT (Model 3). Regarding this latter result, we compared the slopes of the SRT and List Recognition *iSD* scores by computing a *z*-score of the residual difference between their unstandardized slopes. The relative contribution of the SRT *iSD* (*b* = −0.61, *SE* = 0.17) to the prediction of Memory Composite was significantly larger than that of the List Recognition *iSD* (*b* = −0.15, *SE* = 0.07), *z* = −2.50, *p* = 0.01.

The most notable results at the third step, given the aims of the model, were these: SRT *iSD* scores continued to contribute significantly to the prediction of CAMCOG-R Total Score (Model 1) and CAMCOG-R Memory Composite score (Model 3), and CRT *iSD* score continued to contributed significantly to the prediction of CAMCOG-R Memory Composite score (Model 4).

Finally, to examine the influence of measurement timescale on the relative contribution of the different IIV measures to the prediction of cognitive performance, we created a set of models that described how *session-to-session variability* on RT- and accuracy-based tasks predicted (a) CAMCOG-R Total Score, and (b) CAMCOG-R Memory Composite score. For each model, we entered the same demographic factors as in the previous models at the first step, *iSD* RT- and accuracy-based predictors at the second, and mean-based predictors at the third (see Table 5).

**Table 5:** Regression Models: session-to-session reaction time IIV compared to accuracy-based IIV in the prediction of CAMCOG scores (*N* = 48).

**Predictor**	**CAMCOG-R OUTCOME VARIABLE**			
	**Total Score**		**Memory Composite Score**	
	**Model 1**	**Model 2**	**Model 3**	**Model 4**
	**SRT vs. List Recognition**	**CRT vs. List Recognition**	**SRT vs. List Recognition**	**CRT vs. List Recognition**
**STEP 1**				
Group	−0.72^***^	−0.72^***^	−0.84^***^	−0.84^***^
Sex	−0.26^**^	−0.26^**^	−0.26^**^	−0.26^**^
Education	0.23^*^	0.23^*^		
**STEP 2**				
Group	−0.62^***^	−0.66^***^	−0.73^***^	−0.75^***^
Sex	−0.22^**^	−0.25^**^	−0.22^**^	−0.24^**^
Education	0.23^**^	0.22^*^		
***iSD***				
List Recognition	−0.16	−0.09	−0.20^*^	−0.15
SRT/CRT	−0.22^**^	−0.09	−0.18^*^	−0.09
**STEP 3**				
Group	−0.29^**^	−0.29^*^	−0.32^**^	−0.31^**^
Sex	−0.14	−0.15	−0.13	−0.12
Education	0.12	0.12		
***iSD***				
List Recognition	−0.06	−0.01	−0.08	−0.05
SRT/CRT	−0.18^*^	−0.03	−0.12	−0.001
**Mean**				
List Learning	0.07	0.02	0.08	0.04
List Recognition	0.07	0.10	0.23	0.25^*^
Story Memory	0.43^**^	0.46^**^	0.29^*^	0.32^*^
SRT/CRT	0.02	−0.03	−0.03	−0.11

Data presented are β (standard regression coefficient) values. CAMCOG-R, Cambridge Cognitive Examination for Mental Disorders of the Elderly-Revised; SRT, simple reaction time; CRT, choice reaction time.

* p < 0.05,

** p < 0.01,

*** *p < 0.001. All p-values are one-tailed*.

The most notable result at the second step was that, for the RT data, the magnitude of variability decreased markedly from that observed in the trial-to-trial models. For instance, although at Step 2 of the modeling procedure SRT *iSD* was a significant predictor of CAMCOG-R Total Score [Model 1: Δ*R*^2^ = 0.04, *F*_(2, 42)_ = 4.27, *p* = 0.02, Cohen's *f* = 0.20] and of CAMCOG-R Memory Composite score [Model 3: Δ*R*^2^ change = 0.04, *F*_(2, 43)_ = 3.99, *p* = 0.03, Cohen's *f* = 0.18], CRT *iSD* was not a significant predictor of either outcome. Again, List Recognition *iSD* score only predicted Memory Composite score significantly when it was entered together with SRT (Model 3). This time, the relative contributions of SRT *iSD* (*b* = −0.16, *SE* = 0.07) and List Recognition *iSD* (*b* = −0.18, *SE* = 0.08) to the prediction of Memory composite were not significantly different, *z* = −0.22, *p* = 0.59.

The most notable results at the third step were, again, that the predictive power of the RT *iSD* scores decreased markedly from that observed in the trial-to-trial models. Here, the only significant finding, given the aims of the model, was that SRT *iSD* scores continued to contribute significantly to the prediction of CAMCOG-R Total Score (Model 1).

## Discussion

This study provided a direct comparison of the relative sensitivity of reaction time- and accuracy-based estimates of intraindividual variability to cognitive compromise. We systematically replicated findings presented by Hultsch et al. (2000), showing that (a) RT-based measures of IIV differentiated a dementia group from a group of healthy older adults, (b) increasing the timescale of measurement (i.e., measuring on a session-to-session rather than a trial-to-trial basis) reduced the sensitivity of RT-based IIV, and (c) generally, RT-based IIV was a better predictor of cognitive status than accuracy-based IIV, even after adjusting for timescale of measurement. We extended upon previous findings by showing that accuracy-based IIV (a) could also differentiate patients with AD from healthy older adults, (b) correlated significantly with overall cognitive function and episodic memory performance in both patients and controls, and (c) was a significant predictor of episodic memory performance, even after controlling for sex and group status (AD patient vs. control).

Of the accuracy-based IIV measures that formed part of our investigation, only RBANS List Recognition was sensitive to between-group differences, correlated with CAMCOG-R Total Score and Memory Composite score, and predicted performance on the CAMCOG-R Memory Composite variable after controlling for sex and group status. Although Hultsch et al. (2000) also used measures of recognition memory to derive accuracy-based IIV, they found them to have no significant value in distinguishing dementia patients from controls. We argue that this cross-study difference is attributable to sample characteristics: Whereas we used a homogeneous group of patients with AD, Hultsch and colleagues used a heterogeneous clinical group [i.e., some of their patients had been diagnosed with vascular dementia (VaD) and others with AD]. Patients with VaD perform significantly better than those with AD on recognition memory tasks (Tierney et al., 2001; Román et al., 2002). Hence, including both VaD and AD patients in a single clinical group is likely to diminish the sensitivity of a recognition-based measure to neurological compromise.

We suggest, therefore, that accuracy-based IIV measures are useful in detecting neurocognitive impairment, but that there must be a careful match between the type of task from which the IIV measure is derived and the purportedly compromised cognitive domain. In other words, accuracy-based IIV measures have less utility when they are considered as indicators of diffuse cognitive or neurological dysfunction: They are best used as indicators of a specific type of cognitive impairment linked to a specifically damaged neuroanatomical site or system. Murphy et al. (2007) demonstrated this point empirically. They administered parallel forms of a list-learning task eight times over 4 days to young (*M* = 23.4 years) and older (*M* = 73.3 years) adults. The groups were differentiated by accuracy-based IIV scores derived from tasks assessing frontal lobe function (e.g., false memory tests), but not by those derived from tasks assessing medial temporal lobe (MTL) function (e.g., learning, delayed recall). The authors proposed that age-related changes in the integrity of the frontal lobes (changes not typically present in the MTL) explained this finding. These specific structural changes made it much more likely that there would be increased variability in the performance of the older adults relative to the younger counterparts on the frontal tasks, but not the MTL tasks. Another minor empirical demonstration of this regional specificity consideration is that, among the patient group in the present study, the largest magnitude of variability we observed was on the List Recognition task (see Table 2).

Whereas List Recognition *iSD* scores differentiated between patients and controls, and were significantly associated with scores on the outcome measures, no such relationships were observed for the List Learning and Story Memory *iSD*s. Given that performance on all three tests requires participation from neural networks that are centered on the MTL and that are compromised by AD pathology (Traykov et al., 2007; Peña-Casanova et al., 2012), this result is unexpected: In this context, IIV on the three tasks should have been similar.

One possible reason for this unexpected result relates to the differing nature of the processing demands made by the List Learning, Story Memory, and List Recognition tasks. Although all three tasks require the participant to retrieve previously-encoded information, the former two make heavier demands on cognitive resources because they are free recall, and not aided-recall, tasks. In other words, they present no cues to assist retrieval of the learned information, and therefore require more self-generated strategic processing (Moscovitch and Winocur, 1992; Dickerson et al., 2007). Tasks with greater strategic processing demands typically produce higher degrees of score variability, particularly when performance is measured across several learning trials (Allaire and Marsiske, 2005), as was the case with both List Learning and Story Memory. Hence, performance on those subtests may be more vulnerable than List Recognition performance to *adaptive variability* (Li et al., 2004). Because the presence of adaptive variability tends to increase IIV, a confound within the current design is that one of the three memory tasks we used to measure IIV featured lower strategic processing demands than the other two.

Nonetheless, there is clinical value in the finding that an accuracy-based measure of inconsistency can significantly predict episodic memory performance. In the clinic, accuracy-based assessment is far more prevalent than latency-based assessment, and the List Recognition task we used here is a standard element of many clinical neuropsychological test batteries. Of note here, however, is that a current trend in IIV studies that use accuracy-based measures is to move away from operationalizing variability as *inconsistency* across time and toward *dispersion* across tasks (within and across cognitive domains) or across items within a test of global cognition (e.g., CAMCOG-R). Findings from IIV studies using this latter operationalization indicate successful prediction of cognitive decline and clinical dementia status above and beyond mean-level performance (Tractenberg and Pietrzak, 2011; Kälin et al., 2014). Accuracy-based measures of dispersion may be more practical than RT measures of IIV for clinicians as the test from which they are derived are already used frequently within standard neuropsychological test batteries, and they avoid the need for multiple trials of administration (Kälin et al., 2014).

## Limitations

Two limitations of the study's RT-based measures might have reduced their sensitivity to impairment on the cognitive outcomes we sought to measure. The first involves how engaging the RT tasks were for participants. We observed that, for healthy controls, bivariate associations between (a) RT means and CAMCOG-R Total Scores, and (b) variability scores and CAMCOG-R Total Scores were in the opposite direction from what might have been expected (see Supplemental Material). That is, participants with higher CAMCOG-R Total Scores showed slower and more variable performance on both the SRT and the CRT tasks. One explanation is that the repetitive nature of the serial assessments, combined with the relative ease of the RT tasks, may have resulted in a lack of engagement among higher-functioning individuals. This speculation is consistent with research indicating that a lack of task engagement (e.g., due to boredom) during prolonged repetitive tasks may reduce mean RT performance and increase RT variability (Pan et al., 1994; Langner et al., 2010; Garrett et al., 2012; Wang et al., 2014).

A second, and related, limitation involves the regional specificity of the RT measures. Performance on the kinds of SRT and CRT tasks used here activates a complex combination of cognitive control processes (including visual encoding, motor preparation, response selection, and execution), with common neural substrates located largely in the frontal lobes (MacDonald et al., 2000; Lo and Andrews, 2015). As noted above, accuracy-based IIV measures are most useful when the task from which they are derived taps into functioning of the area purportedly compromised in the samples under scrutiny. Such regional specificity considerations may also apply to RT-based IIV measures (MacDonald et al., 2008). Following this line of argument, an RT measure better suited to the purposes of the current study may have been one derived from tasks sensitive to episodic memory function [e.g., the recognition latencies from the list and story tasks used by Hultsch et al. (2000)].

Hence, future research in the field might consider adapting RT tests to make them more engaging, and to ensure that they meet considerations related to regional specificity. Using latency scores from tasks that are typically used to produce accuracy-based outcomes may, in fact, also improve task engagement because participants typically find such tasks more challenging than basic RT tasks (Allaire and Marsiske, 1999).

## Summary and conclusion

We set out to systematically replicate and extend important previous findings regarding the use of intraindividual variability measures in the detection of neurodegenerative disease (Hultsch et al., 2000). Our replication was successful: Results indicated that RT-based IIV measures are superior predictors of cognitive compromise than accuracy-based IIV measures, even after adjusting for timescale of measurement. Our extension was also successful: Results indicated that, by using a homogeneous clinical sample (i.e., early-to-mid-stage Alzheimer's disease patients) and measuring overall cognitive function as well as a performance within a targeted cognitive domain, accuracy-based IIV measures may be useful indicators of underlying pathology. The present study therefore contributes toward understanding the relative utility of RT- and accuracy-based IIV measures in detecting neurocognitive impairment in older adults, and also responds to the AARR call for empirical evaluation of sensitive markers of cognitive change in patients with AD.

## Ethics statement

This study was carried out in accordance with the recommendations of the University of Cape Town Research Ethics Code for Research Involving Human Participants with written informed consent from all subjects. All subjects gave written informed consent in accordance with the Declaration of Helsinki. The protocol was approved by the Research Ethics Committees of the University of Cape Town's Department of Psychology and the Faculty of Health Sciences.

## Author contributions

BC: contributed to the conception and design of the study, participant recruitment and acquisition of data, data analysis and interpretation, drafting of the manuscript, and critical revisions of the manuscript for important intellectual content; KT: contributed to the conception and design of the study and critical revisions of the manuscript for important intellectual content; MC: contributed to participant recruitment and acquisition of data; All three authors (BC, KT, and MC) approved of the final version of the manuscript to be submitted for publication.

### Conflict of interest statement

The authors declare that the research was conducted in the absence of any commercial or financial relationships that could be construed as a potential conflict of interest.
