# Supplementary material for: Both Reaction Time and Accuracy Measures of Intraindividual Variability Predict Cognitive Performance in Alzheimer's Disease
Source: Front Hum Neurosci. 2018 Apr 9;12:124. doi: 10.3389/fnhum.2018.00124 (PMC5900796; doi:10.3389/fnhum.2018.00124)
Supplement: Supplementary file 1 [file DataSheet1.docx]

**Supplemental Material**

Table A1

*Brief Outline of Cognitive Tests*

| Test (battery) | Source |
| --- | --- |
| *n*-back | http://step.psy.cmu.edu/scripts-plus/ |
|  |  |
| Motor Screening (CANTAB) | Fray, Robbins, & Sahakian (1996) |
| Reaction Time* (CANTAB) |  |
| Attention Switching Task (CANTAB) |  |
| Paired Associates Learning (CANTAB) |  |
|  |  |
| Repetitive Timing Task | Duchek, Balota, & Ferraro (1994) |
|  |  |
| List Learning (RBANS) | Randolph (1998) |
| Story Memory (RBANS) |  |
|  |  |
| Finger Tapping (Halstead-Reitan) | Reitan & Davidson (1974) |
|  |  |
| Grooved Pegboard Test | Mathews & Klove (1964) |

*Note*: * Includes both simple- and choice- reaction time tests.

Table A2

*Order of Test Administration*

| Test Order 1* | Test Order 2 | Test Order 3 |
| --- | --- | --- |
|  |  |  |
| *n*-back (block 1) | CANTAB | Grooved Pegboard |
| Repetitive Timing | List Learning – learning | Finger Tapping |
| *n*-back (block 2) | *n*-back (block 1) | List Learning |
| List Learning - Learning | Repetitive Timing | CANTAB |
| Grooved Pegboard | *n*-back (block 2) | List Learning – delay |
| Finger Tapping | List Learning – delay | List Learning – recognition |
| List Learning – delay^1^ | List Learning – recognition | Story Memory – learning |
| List Learning – recognition | Story memory – learning | *n*-back (block 1) |
| Story Memory – learning^1^ | Grooved Pegboard | Repetitive Timing |
| CANTAB^2^ | Finger Tapping | *n*-back (block 2) |
|  |  |  |

*Note*: *Due to laboratory constraints the sequence of the test order (i.e. which test order was administered first, second, or third) had to be varied to accommodate concurrent assessments. ^1^The delay condition was administered after the prescribed timed interval. ^2^The CANTAB tests were administered consistently in the following order: Motor Screening, Reaction Time (block 1), Paired Associates Learning, Attention Switching, and Reaction Time (block 2).

Table A3

*Fixed Effects from Random Intercept Models (N = 48)*

|  | SRT | | CRT | | SRT by session | | CRT by session | | List Learning | | List Recognition | | Story Memory | |
| --- | --- | --- | --- | --- | --- | --- | --- | --- | --- | --- | --- | --- | --- | --- |
| Variable | *F* | *p* | *F* | *p* | *F* | *p* | *F* | *p* | *F* | *p* | *F* | *p* | *F* | *p* |
| Test Order | 1.37 | .26 | 9.45 | < .001*** | 0.19 | .82 | 1.67 | .19 | --- | --- | --- | --- | --- | --- |
| Blocks | 15.40 | < .001*** | 18.45 | < .001*** | --- | --- | --- | --- | --- | --- | --- | --- | --- | --- |
| Trials/Session | 0.50 | .48 | 0.71 | .40 | 2.08 | .15 | 6.94 | .01* | 0.06 | .81 | 10.27 | .002** | 10.55 | .002** |
| Group | 7.49 | .01* | 10.58 | .002** | 7.52 | .009** | 10.58 | .002** | 32.87 | < .001*** | 47.85 | < .001*** | 35.38 | < .001*** |
| Sex | 5.96 | .02* | 7.39 | .01* | 5.98 | .02* | 7.40 | .01* | 0.08 | .78 | 0.07 | .80 | 5.70 | .02* |
| Income^b^ | 0.76 | .56 | 0.45 | .77 | 0.77 | .56 | 0.46 | .77 | 0.40 | .80 | 0.28 | .89 | 0.78 | .55 |
| Age | 0.06 | .81 | 0.01 | .98 | 0.06 | .81 | 0.001 | .98 | 2.03 | .16 | 0.58 | .45 | 0.08 | .78 |
| Education^a^ | 0.30 | .59 | 0.77 | .39 | 0.304 | .59 | 0.77 | .39 | 1.92 | .17 | 0.30 | .59 | 4.96 | .03* |

*Note.* SRT = simple reaction time; CRT = choice reaction time. ^a^Highest level of education attained. ^b^Monthly household income. **p* < .05. ** *p* < .01. *** *p* < .001. All *p*-values are two-tailed.

Table A4

*Bivariate Correlations within Control Group: Predictor and outcome variables (N = 25)*

|  | CAMCOG-R Outcome Variable | |
| --- | --- | --- |
| Predictor Variable | Total Score | Memory Composite |
| *iSD* |  |  |
| SRT | .33 | -.08 |
| CRT | .19 | -.19 |
| List Learning | -.21 | -.04 |
| List Recognition | -.04 | -.23 |
| Story Memory | -.43* | .01 |
| Mean |  |  |
| SRT | .32 | .25 |
| CRT | .30 | .18 |
| List Learning | .33 | .53** |
| Recognition | .36 | .33 |
| Story Memory | .64** | .54** |

*Note*. CAMCOG-R = Cambridge Cognitive Examination for Mental Disorders of the Elderly-Revised. *iSD* = intraindividual standard deviation; SRT = simple reaction time; CRT = choice reaction time. **p* < .05. ** *p* < .01. All *p*-values are one-tailed.

Table A5

*Bivariate Correlations with AD Patient Group: Predictor and outcome variables (N = 23)*

|  | CAMCOG-R Outcome Variable | |
| --- | --- | --- |
| Predictor Variable | Total Score | Memory Composite |
| *iSD* |  |  |
| SRT | -.51** | -.52** |
| CRT | -.39* | -.51** |
| List Learning | .02 | -.11 |
| List Recognition | -.20 | -.24 |
| Story Memory | -.01 | -.11 |
| Mean |  |  |
| SRT | -.36* | -.41* |
| CRT | -.39* | -.48* |
| List Learning | .55** | .56** |
| Recognition | .34 | .52* |
| Story Memory | .79** | .73** |

*Note*. CAMCOG-R = Cambridge Cognitive Examination for Mental Disorders of the Elderly-Revised. *iSD* = intraindividual standard deviation; SRT = simple reaction time; CRT = choice reaction time. **p* < .05. ** *p* < .01. All *p*-values are one-tailed.
